# Mutations in the *ubiA* gene are the major mechanism of ethambutol resistance in *Mycobacterium avium*

**DOI:** 10.1128/spectrum.03151-25

**Published:** 2026-03-23

**Authors:** Yuwei Qiu, Xu Dong, Dan Cao, Xiuzhi Jiang, Zhongkang Ji, Pusheng Xu, Yi Li, Kaijing Xu, Ying Zhang

**Affiliations:** 1 State Key Laboratory for Diagnosis and Treatment of Infectious Diseases, National Clinical Research Center for Infectious Diseases, China-Singapore Belt and Road Joint Laboratory on Infection Research and Drug Development, National Medical Center for Infectious Diseases, Collaborative Innovation Center for Diagnosis and Treatment of Infectious Diseases, The First Affiliated Hospital, Zhejiang University School of Medicine 26441, Hangzhou, China; 2 Institute for Persistent Infections and Antibiotic Resistance, Dongguan Innovation Institute, School of Medical Technology, Guangdong Medical University 12453, Zhanjiang, China; Rutgers New Jersey Medical School, Newark, New Jersey, USA

**Keywords:** *Mycobacterium avium*, ethambutol, resistance mechanism, mutations, *ubiA*, minimum inhibitory concentration

## Abstract

*Mycobacterium avium-intracellulare* complex (MAC) infections are the most common nontuberculous mycobacterial infections, and ethambutol (EMB) is one of the main therapeutic agents used to treat MAC infections. However, the EMB resistance profile and its resistance mechanisms in *M. avium* remain poorly understood. This study determined the minimum inhibitory concentration (MIC) of 40 *M. avium* clinical strains, revealing that 97.5% (39/40) of the strains were intermediate (MIC = 4 µg/mL) or resistant (MIC ≥ 8 µg/mL) to EMB. One susceptible clinical isolate strain 245 (MIC = 2 µg/mL) was picked for EMB-resistant mutant isolation, and a total of 121 resistant mutants were isolated and subjected to whole-genome sequencing or Sanger sequencing. Integrated analysis revealed that 94.21% (114/121) of the mutants carried mutations in the *ubiA* gene, which encodes decaprenylphosphoryl-β-D-5-phosphoribose (DPPR) synthase—an enzyme involved in cell wall biosynthesis that has been associated with high-level EMB resistance in *Mycobacterium tuberculosis*. Complementation with the wild-type *ubiA* gene restored EMB susceptibility in EMB-resistant mutants and clinical strain 322 (EMB MIC = 64 µg/mL) with *ubiA* mutation, reducing the MICs from 32 to 4 μg/mL and from 64 to 8 μg/mL, respectively. This study indicates that the *ubiA* mutation is the major mechanism of EMB resistance in *M. avium*, which should facilitate the development of molecular tests for rapid detection of resistance in this organism.

**IMPORTANCE:**

This study identified *ubiA* as a key gene associated with ethambutol (EMB) resistance in *Mycobacterium avium*, a finding which has not previously been reported. Furthermore, although *ubiA* has been linked to EMB resistance in *Mycobacterium tuberculosis* (MTB), mutations in this gene account for only a small proportion of EMB-resistant MTB isolates. In contrast, our study showed that *ubiA* played a major role in EMB resistance in *M. avium*. Our findings contribute to the development of molecular assays for rapid detection of EMB resistance in *M. avium* and highlight the distinct main resistance mechanisms across different bacterial species to the same antibiotic.

## INTRODUCTION

*Mycobacterium avium* complex (MAC) is a group of several slow-growing mycobacteria and is commonly found in the environment, such as water and soil (1, 2). MAC is primarily transmitted through aerosol inhalation and can cause infections, often in immunocompromised individuals (3). Patients who are aged, immunosuppressed, or have other respiratory diseases (such as cystic fibrosis, bronchiectasis, and COPD) are at higher risk of developing severe clinical symptoms following MAC infections (4–6). MAC comprises various subspecies, including *M. avium*, *M. intracellulare*, *M. colombiense, M. timonense*, etc., with *M. avium* being one of the most prevalent species globally (7). Current treatment guidelines for MAC infections recommend a multi-drug combination therapy with macrolides (clarithromycin/azithromycin) as the core drug, along with ethambutol (EMB), rifampicin (RIF), aminoglycosides or other drugs, and a three-drug regimen (macrolide, RIF, and EMB) is commonly recommended for the treatment of *M. avium* complex pulmonary disease (8).

EMB, as a key component in MAC treatment regimens, inhibits bacterial growth by interfering with arabinogalactan biosynthesis in the mycobacterial cell wall (9). Not only does its use significantly improve sputum conversion rates compared to RIF, streptomycin, or fluoroquinolones in the treatment of CLR-resistant MAC infections, but it also effectively prevents the development of macrolide resistance (10, 11). In patients with AIDS, EMB also had greater antibacterial activity than either RIF or clofazimine, supporting its unshakable position in the treatment of MAC infection (12). In terms of drug susceptibility, although a previous study classified MAC strains as susceptible (minimum inhibitory concentration [MIC] ≤ 2 µg/mL), intermediate (MIC = 4 µg/mL), or resistant (MIC ≥ 8 µg/mL) to EMB, many studies indicated that the MICs of *M. avium* strains of EMB are mainly 8 μg/mL, while the Clinical and Laboratory Standards Institute (CLSI) does not yet have EMB susceptibility or resistance breakpoints for *M. avium* (13–15). Furthermore, it has been suggested that when MAC isolates exhibit MICs ≥8 µg/mL for both EMB and RIF, the clinical treatment success rate will decrease significantly, indicating the negative correlation between successful treatment outcome with resistance to EMB and RIF (16). Therefore, elucidating the mechanism of EMB resistance in MAC remains clinically significant. To date, *embB/embR aftA* and *ubiA* genes have been proven to be associated with EMB resistance in *Mycobacterium tuberculosis* (MTB) (17–20), but the resistance mechanism in MAC is far less explored. Although previous studies have revealed that *embA*/*B* contribute to elevated EMB resistance in *M. avium* (21), clinical investigation indicated that *embB* mutations account for only about 4% of the EMB-resistant MAC clinical isolates (22), indicating other major EMB resistance mechanisms remain to be identified.

To better understand the mechanisms of EMB resistance in *M. avium*, here, we isolated EMB-resistant mutants and performed whole-genome sequencing and Sanger sequencing to investigate the potential EMB resistance mechanisms in *M. avium*.

## MATERIALS AND METHODS

### Strains and antibiotics

All the *M. avium* clinical strains were provided by The First Affiliated Hospital, Zhejiang University School of Medicine (Hangzhou, China). Clinical strains were identified as *M. avium* through whole-genome sequencing (Uni-medica, Shenzhen, China). Ethambutol (Aladdin, Shanghai, China) was dissolved in DMSO (Aladdin, Shanghai, China) and prepared as a 5.12 mg/mL stock solution and was stored at −20°C before use. Kanamycin sulfate (Aladdin, Shanghai, China) was dissolved in double-distilled water and prepared as a 100 mg/mL stock solution, and was stored at −20°C before use.

### MIC determination and mutant screening

The MIC determination was performed using the microdilution method as previously described (23). The drug solution was diluted to 128 µg/mL in Middlebrook 7H9 broth (Difco, USA) supplemented with 5% OADC (oleic acid, albumin, glucose, and catalase) and 0.2% glycerol, and serial twofold dilutions (100 μL per well) were performed in 96-well plates (NEST, Wuxi, China). The bacterial suspension was diluted to 0.5 MacFarland Standard in Middlebrook 7H9 broth as mentioned above at first, then further diluted 100-fold, and a 100 μL aliquot of the suspension was added into each well with EMB, resulting in 11 drug concentrations ranging from 64 to 0.0625 μg/mL. The final bacterial count per well was approximately 5 × 10⁵ CFU/mL (ranging from 1 × 10⁵ to 1 × 10^6^ CFU/mL). Positive control wells that contained only bacteria were set in the last column of the 96-well plates, and negative control wells that contained only the drug were set separately. The plates were incubated at 37°C for 2 weeks, and the results were read. The MIC test was performed in triplicate.

After MIC determination in 7H9 broth, the strain used for isolating EMB-resistant mutant strains was identified. Middlebrook 7H11 agar plates (Hopebio, Qingdao, China, containing 10% OADC and 0.5% glycerol) with EMB concentrations ranging from 1/4 to 4 times MIC were prepared, along with an antibiotic-free 7H11 agar plate as a control. The experimental approach for determining MIC on agar plates was described previously (24). A 20 μL of 1,000-fold diluted log-phase bacterial suspension was added to the 7H11 agar plates to confirm the MIC was consistent in both liquid and solid media. After 2 weeks of incubation at 37°C, the MIC on agar plates was determined.

For mutant screening, based on the MIC results from the 7H11 plates, 7H11 plates containing EMB at 4, 8, 16, and 32 times the MIC were prepared. A 100 μL aliquot of the stationary-phase bacterial suspension was then spread on the 7H11 EMB plates and incubated for 2 weeks to determine the optimal concentration to screen the EMB-resistant strains, which was used in the follow-up mutant screening. Single colonies of resistant mutants were picked and streaked onto 7H11 plates with a higher concentration of EMB, followed by incubation for another 2 weeks to make sure that the bacteria were resistant to EMB. The original sensitive strain was streaked onto the plate as a control.

### Whole-genome sequencing

Genomic DNA was isolated from 10 mL of bacterial culture, and a number of resistant mutants and the original sensitive strain were picked. The cultures were centrifuged at 4,000 rpm for 10 min at 4°C, after which the supernatant was discarded. The pellets were washed twice with sterile water. The samples were sent for whole-genome sequencing using the Illumina platform (Novogene, Beijing, China). The genomic sequences of the resistant mutants were compared to those of the sensitive parent strain, and single-nucleotide polymorphisms (SNPs) were analyzed using software Snippy to identify putative mutations associated with EMB resistance in *M. avium*.

### PCR and Sanger sequencing

The *ubiA* gene sequence was obtained from the whole-genome sequencing data of the susceptible strain by bacterial genome annotation software Bakta. Primers were designed as follows: ubiA_F: ATGACCGAGGAAACGCAGG; ubiA_R: CTAACCGAAGGCAACGGCG. Phanta Flash Master Mix (Dye Plus) (Vazyme, Nanjing, China) was used for PCR amplification, and the PCR conditions were as follows: denaturation at 98°C for 10 min, followed by 35 cycles of 98°C for 10 s, 60°C for 5 s, and 72°C for 5 s, with a final extension at 72°C for 1 min.

For all remaining resistant mutants, the original susceptible strain and all other clinical strains, a 918-base pair fragment containing the *ubiA* gene was subjected to Sanger sequencing (Tsingke, Beijing, China), and the sequences were aligned for comparison by SnapGene.

### Gene complementation experiment

The target wild-type *ubiA* gene was amplified by PCR using the following primers: pmvubiA-F: caggaattcgatatcaagcttATGACCGAGGAAACGCAGG; pmvubiA-R: acgctagttaactacgtcgacCTAACCGAAGGCAACGGCG. The PCR product was purified by Fastpure Gel DNA Extraction Mini Kit (Vazyme). The pMV306hsp plasmid (25, 26) was digested with *Hind*III and *Sal*I, followed by cloning of the *ubiA* PCR product into the linearized plasmid vector using ClonExpress Ultra One Step Cloning Kit (Vazyme). The recombinant plasmid was then transformed into *Escherichia coli* DH5α (Vazyme) by heat shock, and the transformant was incubated in a shaker at 37°C for 1 h, followed by plating onto LB agar plates (Hopebio, Qingdao, China) containing 100 µg/mL kanamycin and incubated at 37°C overnight. Single colonies were selected the next day, cultured in LB broth (Hopebio, Qingdao, China) supplemented with 100 µg/mL kanamycin, and verified by Sanger sequencing to confirm that the wild-type *ubiA* gene was successfully cloned into pMV306hsp. The wild-type *ubiA* recombinant plasmid was then extracted with the Fastpure Plasmid Mini Kit (Vazyme) and electroporated (2,500 V, 1,000 Ω, and 25 uF) into the EMB-resistant strains. The electroporated mutant strains were incubated in a 37°C shaker overnight and then plated onto 7H11 agar plates containing 100 µg/mL kanamycin and incubated at 37°C for 2 weeks. Single colonies that grew on the plates were then subjected to PCR sequencing to confirm that the wild-type *ubiA* gene was successfully introduced into the EMB-resistant strains. The complementation strains were tested for EMB susceptibility in 7H9 broth to validate the association between *ubiA* mutation and EMB resistance. Empty vector controls were included to exclude the potential effects of vector on MIC determinations.

## RESULTS

### Determination of EMB MICs for *M. avium* clinical strains

The EMB MIC values for 40 *M. avium* clinical strains were determined as described in the Materials and Methods. As summarized in Table 1, according to the previous study (13), only one clinical strain (strain 245) was susceptible to EMB (MIC ≤ 2 µg/mL), while all other clinical strains exhibited at least intermediate susceptibility (MIC ≥ 4 µg/mL) to EMB (97.5%, 39/40), and 82.5% of the clinical strains (33/40) were resistant (MIC ≥ 8 µg/mL) to EMB (13).

**TABLE 1:** Ethambutol MIC distribution of *M. avium* clinical isolates

Strain name	MIC (µg/mL)	Source	Total
245	2	Sputum	1
118	4	Sputum	6
458	4	Sputum	
141	4	Sputum	
150	4	Sputum	
635	4	Sputum	
1055	4	Urine	
1006	8	Sputum	21
640	8	Sputum	
990	8	Sputum	
1237	8	Sputum	
1004	8	Sputum	
515	8	Sputum	
1224	8	Sputum	
142	8	Sputum	
715	8	BALF^*a*^	
1200	8	Drainage fluid	
938	8	Sputum	
1221	8	Sputum	
642	8	Sputum	
879	8	Sputum	
643	8	Sputum	
123	8	Sputum	
375	8	Sputum	
658	8	Sputum	
757	8	Sputum	
1015	8	Sputum	
1126	8	Sputum	
1083	16	Drainage fluid	10
1148	16	Sputum	
758	16	Secretion	
158	16	Sputum	
765	16	Sputum	
699	16	Sputum	
166	16	Sputum	
119	16	Sputum	
304	16	Hydrothorax	
374	16	Sputum	
860	32	Drainage fluid	1
322	64	Sputum	1
Total			40

^*a*^ BALF, bronchoalveolar lavage fluid.

### Mutant isolation

From the above results, we selected the EMB-susceptible strain 245 (EMB MIC = 2 µg/mL) for isolating EMB-resistant mutants. Since the isolation of mutant strains was performed on 7H11 plates, the MIC of clinical strain 245 was reassessed on 7H11 agar plates containing EMB at concentrations ranging from 0.5 to 8 μg/mL, based on the previous MIC result obtained in 7H9 broth. The final EMB MIC of strain 245 on 7H11 agar plate was determined to be 4 μg/mL, which is twofold higher than its MIC in 7H9 liquid medium.

Based on the MIC results obtained from 7H11 agar plates, mutant isolation was conducted on 7H11 agar plates containing EMB at concentrations of 4× MIC and above, i.e., 16, 32, 64, and 128 μg/mL. After 2 weeks of incubation, it was determined that 16 μg/mL was the optimal concentration for mutant isolation, as 7H11 agar plates containing higher EMB concentrations failed to grow mutant colonies. The concentration of 16 μg/mL was subsequently used for large-scale screening of EMB-resistant mutants. All the colonies that grew on the plates containing 16 μg/mL were picked and streaked onto 7H11 agar plates containing EMB at 32 μg/mL together with the original susceptible strain. The mutants that grew both on plates containing 16 and 32 μg/mL EMB were regarded as authentic resistant mutants, and finally, a total of 121 mutants were successfully isolated.

### Whole-genome sequencing and Sanger sequencing

To identify the potential mechanisms of EMB resistance in *M. avium*, whole-genome sequencing was performed initially on eight EMB-resistant mutants along with the parent susceptible strain as a control. The results showed that all the mutants harbored mutations in the *ubiA* gene, strongly implying an association between *ubiA* gene mutation and EMB resistance in *M. avium*. The remaining 113 resistant mutants were all subsequently subjected to *ubiA* Sanger sequencing. The results showed that most of the mutants harbored mutations in *ubiA* (93.81%, 106/113), which is consistent with and supports the initial whole-genome sequencing findings. Integrating the results from both whole-genome and Sanger sequencing, the mutations were found to be distributed throughout the *ubiA* gene. The majority of mutants carried single-site point mutations at different locations of the *ubiA* gene, accounting for 80.99% (98/121) of the observed mutations. However, 13.22% (16/121) of the mutants had multi-site mutations, insertions, and deletions in the *ubiA* gene (Table 2). Additionally, seven mutants (5.79%, 7/121) exhibited no mutations in the *ubiA* gene. To investigate other potential EMB resistance mechanisms in *M. avium*, we further performed whole-genome sequencing on these seven mutants lacking *ubiA* mutations. However, comparative genomic analysis revealed no mutations relative to the parent strain, implicating that their resistance mechanism remains unknown. The results of the *ubiA* mutations identified through both whole-genome sequencing and Sanger sequencing are summarized in Table 2.

**TABLE 2:** The proportion of *ubiA* mutations at different sites in all EMB-resistant mutants^*a*^

Nucleotide mutation	Mutant type	Amino acid change	Number of the mutants	Proportion (%)
C113A	SNP	Ala38Glu	2	1.65
G121C	SNP	Ala41Pro	6	4.96
G124C	SNP	Ala42Pro	3	2.48
T128C	SNP	Leu43Pro	7	5.79
C495G	SNP	Ile165Met	1	0.83
G503A	SNP	Gly168Asp	7	5.79
T524C	SNP	Leu175Pro	6	4.96
A526G	SNP	Thr176Ala	1	0.83
C527T	SNP	Thr176Ile	10	8.26
A530T	SNP	Gln177Leu	1	0.83
T535C	SNP	Phe179Leu	15	11.57
T535A	SNP	Phe179Ile	1	0.83
T536C	SNP	Phe179Ser	3	2.48
T536G	SNP	Phe179Cys	1	0.83
C537G	SNP	Phe179Leu	5	4.13
C537A	SNP	Phe179Leu	2	1.65
T542C	SNP	Leu181Pro	9	7.44
T542A	SNP	Leu181Gln	1	0.83
T722C	SNP	Phe241Ser	5	4.13
T722G	SNP	Phe241Cys	2	1.65
G728A	SNP	Arg243His	7	5.79
G754T	SNP	Val252Leu	2	1.65
G754C	SNP	Val252Leu	1	0.83
T518A and C520G	MNP	Ile173Asn and Arg174Gly	1	0.83
Ins of AAC 527	Insertion	FSC176	1	0.83
Del of 100_102 CTG	Deletion	FSC34	1	0.83
Del of 109_111 CTG	Deletion	FSC37	2	1.65
Del of 115_123 CCGGTGGCG	Deletion	FSC39	1	0.83
Del of 118_126 GTGGCGGCG	Deletion	FSC40	1	0.83
Del of 124_126 GCG	Deletion	FSC42	6	4.96
Del of 151_153 TAC	Deletion	FSC51	1	0.83
Del of 757_759 GTG	Deletion	FSC253	2	1.65
No mutations			7	5.79
Total mutants			121	

^*a*^ Ins, insertion; Del, deletion; FSC, frame shift codon; SNP, single nucleotide polymorphism; MNP, multiple nucleotide polymorphism.

### Majority of *M. avium* clinical isolates resistant to EMB carry mutations in *ubiA* gene

To assess whether mutations in *ubiA* are associated with EMB resistance in *M. avium* clinical strains, we performed Sanger sequencing of the *ubiA* gene on the remaining 39 *M. avium* clinical strains (Table 1) and compared their sequences with that of strain 245, the EMB-susceptible strain. Sequencing results revealed nonsynonymous *ubiA* mutations in 32 of 39 (82.05%) clinical strains, while seven strains (six strains with EMB MIC ≥ 8 µg/mL, one strain with MIC = 4 µg/mL) (7 of 39 = 17.95%) did not have *ubiA* mutations. Among the 32 strains with *ubiA* mutations, 27 were resistant to EMB (MIC ≥ 8 µg/mL) and five exhibited intermediate susceptibility (MIC = 4 µg/mL), suggesting a correlation between *ubiA* variations and EMB susceptibilities in different *M. avium* clinical strains. The Sanger sequencing results for the *ubiA* gene in clinical strains are summarized in Table 3.

**TABLE 3:** Mutations in the *ubiA* gene of *M. avium* clinical strains compared to the EMB susceptible strain 245

Strain name	Number of strains	Nucleotide variations	Amino acid changes
1126, 118, 141, 635, 1055, 1006, 640, 990, 1237, 1004, 515, 142, 715, 1200, 642, 643, 123, 375, 658, 1015, 1083, 1148, 758, 158, 765, 166, 304, 374, 322	29	A169C	Lys57Gln
1126, 118, 141, 635, 1055, 1006, 640, 990, 1237, 1004, 515, 142, 715, 1200, 642, 643, 123, 375, 658, 1015, 1083, 1148, 758, 158, 765, 166, 304, 374, 322	29	G297A	Val99Val
1126, 118, 141, 150, 635, 1055, 1006, 640, 990, 1237, 1004, 515, 142, 715, 1200, 642, 643, 123, 375, 658, 757, 1015, 1083, 1148, 758, 158, 765, 166, 119, 304, 374, 322	32	A333G	Leu111Leu
1126, 118, 141, 150, 635, 1055, 1006, 640, 990, 1237, 1004, 515, 142, 715, 1200, 642, 643, 123, 375, 658, 757, 1015, 1083, 1148, 758, 158, 765, 166, 304, 374, 322	31	C525G	Leu175Leu
1126, 118, 141, 150, 635, 1055, 1006, 640, 990, 1237, 1004, 515, 142, 715, 1200, 642, 643, 123, 375, 658, 757, 1015, 1083, 1148, 758, 158, 765, 166, 119, 304, 374, 322	32	A766G	Ile256Val
119	1	G282C	Pro94Pro
658, 1126, 158	3	A451G	Met151Val
119	1	G711C	Gly237Gly
119	1	G816C	Gly272Gly
757, 150	2	C822T	Ala274Ala
938, 879, 1224, 1221, 860, 699, 458	7	No mutations	

### Complementation of the EMB-resistant mutant with the wild-type *ubiA* restored EMB susceptibility

To determine whether the identified mutations in the *ubiA* gene contribute to EMB resistance, we subsequently introduced the wild-type *ubiA* gene into the randomly selected EMB-resistant mutants. Mutant strain 1 (mutation: G124C) and Mutant strain 19 (mutation: G754C) were used in the gene complementation experiment, with the empty vector being included as a control. The complemented mutants exhibited significantly reduced EMB MIC values (4 μg/mL) compared to mutants transformed with an empty vector control (32 μg/mL) (Table 4). To determine whether the role of *ubiA* in EMB resistance is consistent across different genetic backgrounds, we further electroporated the wild-type *ubiA* gene from strain 245 into strain 322, which exhibited the highest EMB MIC (64 μg/mL) among the clinical strains tested in our study (Table 1). Complementation with the wild-type *ubiA* gene also restored EMB susceptibility, reducing the EMB MIC in strain 322 from 64 to 8 μg/mL (Table 4). These results indicated that mutations in the *ubiA* gene are directly responsible for EMB resistance in diverse *M. avium* strains.

**TABLE 4:** MICs of *M. avium* parent strain and *ubiA* mutant strains complemented with the wild-type *ubiA* gene

Strain name	EMB MIC (µg/mL)
Parent strain 245	2
Mutant strain 1 (*ubiA* mutation: G124C)	32
Mutant strain 1 (pMV306hsp vector alone)	32
Mutant strain 1 (pMV306hspWT*ubiA*)	4
Mutant strain 19 (*ubiA* mutation: G754C)	32
Mutant strain 19 (pMV306hsp vector alone)	32
Mutant strain 19 (pMV306hspWT*ubiA*)	4
Strain 322 (*ubiA* mutations: A169C, A766G)	64
Strain 322 (pMV306hsp vector alone)	64
Strain 322 (pMV306hspWT*ubiA*)	8

## DISCUSSION

Although the use of EMB in the treatment of MAC infections is now widely accepted, the mechanisms underlying EMB resistance in this pathogen remain poorly characterized (8). In this study, we screened 121 EMB-resistant mutants of *M. avium* and performed whole-genome sequencing and targeted *ubiA* Sanger sequencing to investigate the genetic basis of resistance of *M. avium* to EMB. Integrated analysis of both whole-genome sequencing and *ubiA* sequencing data revealed that 94.21% (114/121) of the EMB-resistant strains harbored mutations in the *ubiA* gene, indicating that mutations in *ubiA* are the major mechanism of EMB resistance in *M. avium*. Furthermore, genetic complementation experiments confirmed that *ubiA* is the causative of EMB resistance in *M. avium*.

*ubiA*, which encodes decaprenylphosphoryl-β-D-5-phosphoribose (DPPR) synthase and corresponds to *Rv3806c* in *M. tuberculosis*, is involved in the synthesis of decaprenylphosphoryl-β-D-arabinose (DPA). DPA serves as the donor substrate for arabinosyltransferases, including EmbB and EmbC, which are involved in catalyzing the transfer of arabinose from DPA to form the arabinan components of the mycobacterial cell wall (19, 27). Previous studies indicate that in strains with a wild-type *ubiA* genotype, EMB inhibits EmbCAB activity, thereby disrupting cell wall integrity and resulting in bacterial death (28, 29). However, when *ubiA* mutates, *ubiA* mutations will increase DPA levels, and the elevated DPA will functionally counteract EMB inhibition by providing an excess of the substrate for the EmbCAB, reducing EMB inhibition and conferring high-level resistance in MTB (20). Given the close phylogenetic relationship between *M. avium* and MTB, it is plausible that *ubiA* mutations modulate EMB susceptibility through similar mechanisms in both species. Nevertheless, a striking aspect of our findings is that *M. avium* and MTB acquire EMB resistance through mutations in different genes, despite using the same arabinan biosynthetic pathway. In MTB, resistance is overwhelmingly mediated by mutations in *embB*, which encodes the arabinosyltransferase directly bound and inhibited by EMB (30, 31). In MTB, *ubiA* mutations actually account for a relatively small proportion of EMB-resistant strains, and *embB* mutations are far more common (32). Even in high-level EMB-resistant MTB strains only, the proportion of *ubiA* mutation occurrence fluctuates with changes in geographical location but mostly does not exceed 10%, especially in Asian countries (33–36). In contrast, our results demonstrate that *M. avium* acquires resistance primarily through mutations in *ubiA*, a gene encoding DPPR, which produces the precursor (DPPR/DPA) required for arabinan synthesis, as mentioned above. Unlike MTB, *ubiA* mutations were identified in more than 90% of EMB-resistant *M. avium* mutants selected in this study, and 82.05% (32/39) of the *M. avium* clinical strains, which were all EMB-intermediate/resistant, also harbored *ubiA* mutations, further demonstrating a fundamental clinical difference in EMB resistance between the two species. This species difference likely reflects distinct pathway bottlenecks and metabolic flux control points in the two organisms. In MTB, direct modification of the EMB target (EmbB) is the most effective and lowest-cost route to resistance. However, in *M. avium*, mutations that increase precursor (DPA) production appear to be strongly favored: elevated DPA levels can competitively displace EMB from EmbB/EmbC, reducing drug efficacy even when the target remains wild type. Thus, although the two organisms share the same cell-wall arabinan pathway, the adaptive landscape of EMB resistance differs, with MTB selecting for target modification, and *M. avium* selecting for altered precursor supply. Future comparative studies are needed to elucidate the different antibiotic resistance mechanisms across bacterial species, which could inform the development of species-specific molecular diagnostics.

Apart from the difference in the primary resistance mechanism to EMB between the MTB and *M. avium*, it is noteworthy that seven EMB-resistant mutants in this study lacked mutations in the *ubiA* gene. Furthermore, Sanger sequencing of the *ubiA* gene in all 39 clinical strains revealed that seven clinical strains, six of which exhibited EMB MICs ≥8 µg/mL and one exhibited 4 μg/mL (Tables 1 and 3), had no *ubiA* mutations compared with the susceptible control strain 245 (MIC = 2 µg/mL). These findings strongly suggest the existence of alternative, as yet unidentified resistance mechanisms in *M. avium*, which warrant further investigations.

It is also worth noting that in our MIC assays, nearly all clinical strains exhibited resistance (MIC ≥ 8 µg/mL) or intermediate susceptibility (MIC = 4 µg/mL) to EMB, based on breakpoints established in an earlier study (13). Our MIC test results are consistent with the previous literature, enhancing the reliability of our results (37). This finding mentioned above stands in sharp contrast to the resistance profile observed in *M. tuberculosis*, where only 5.7% of newly diagnosed patients and 17.2% of re-treated patients show EMB resistance under the CLSI guidelines, which define susceptibility as MIC ≤2 µg/mL and resistance as MIC ≥8 µg/mL (38–40). The discrepancy of the EMB resistance rate between MTB and *M. avium* suggests that wild-type *M. avium* may commonly possess intrinsic resistance mechanisms against EMB, or the EMB’s susceptibility/resistance breakpoint for *M. avium* should be modified.

Another interesting point, although not associated with drug resistance, is that mutations in the *ubiA* gene do not confer antibiotic resistance to *M. abscessus* (including EMB) but can trigger a greater pro-inflammatory response (41). Concurrently, these mutations help the bacterium evade immune cell uptake—an effect that is dependent on both the bacterial morphotype and the type of immune cell—and promote biofilm formation, which collectively facilitates the establishment of chronic lung infection (41). Whether *ubiA* mutations play a similar role in establishing a chronic infection by *M. avium* remains to be determined, and it warrants future studies to comprehensively understand the potential diverse effects of *ubiA* on *M. avium* infection.

This study has several limitations. Firstly, as the *ubiA* gene is primarily associated with high-level EMB resistance, and all mutants in this study were selected on 7H11 agar plates containing 32 µg/mL EMB, our approach may have limited the detection of mutations conferring low-level EMB resistance. Secondly, most of the work in our study was conducted using a single clinical strain, and future studies should include a broader collection of EMB-resistant clinical isolates to further examine the correlation between *ubiA* mutations and EMB resistance in *M. avium*. Thirdly, we did not further investigate the resistance mechanisms of EMB-resistant clinical strains without *ubiA* gene mutations due to their different genetic backgrounds, making it difficult to identify the resistance mutations.

In conclusion, our study revealed that, unlike MTB, where *embB* is the main resistance determinant*, ubiA* mutations are the major mechanism of EMB resistance in *M. avium*, which has not been reported before. The finding that *ubiA* mutations are the primary mechanisms of EMB resistance in *M. avium* has implications for the rapid detection of EMB-resistant *M. avium* strains in the future. Further studies are still needed to investigate the alternative mechanisms of EMB resistance in *M. avium*.

## Data Availability

The whole-genome sequence data for the original strain 245 and its 8 mutant strains with *ubiA* mutations that support the findings of this study are submitted to the NCBI database with BioProject ID PRJNA1417368.

## References

[1] Lahiri A, Kneisel J, Kloster I, Kamal E, Lewin A. 2014. Abundance of Mycobacterium avium ssp. hominissuis in soil and dust in Germany - implications for the infection route. Lett Appl Microbiol 59:65–70. doi:10.1111/lam.12243 24612016

[2] Whiley H, Keegan A, Giglio S, Bentham R. 2012. Mycobacterium avium complex--the role of potable water in disease transmission. J Appl Microbiol 113:223–232. doi:10.1111/j.1365-2672.2012.05298.x 22471411

[3] Thomson R, Tolson C, Carter R, Coulter C, Huygens F, Hargreaves M. 2013. Isolation of nontuberculous mycobacteria (NTM) from household water and shower aerosols in patients with pulmonary disease caused by NTM. J Clin Microbiol 51:3006–3011. doi:10.1128/JCM.00899-13 23843489 PMC3754680

[4] Heidary M, Nasiri MJ, Mirsaeidi M, Jazi FM, Khoshnood S, Drancourt M, Darban-Sarokhalil D. 2019. Mycobacterium avium complex infection in patients with human immunodeficiency virus: a systematic review and meta-analysis. J Cell Physiol 234:9994–10001. doi:10.1002/jcp.27859 30548598

[5] Honda JR, Knight V, Chan ED. 2015. Pathogenesis and risk factors for nontuberculous mycobacterial lung disease. Clin Chest Med 36:1–11. doi:10.1016/j.ccm.2014.10.001 25676515

[6] Daley CL. 2017. Mycobacterium avium complex disease. Microbiol Spectr 5. doi:10.1128/microbiolspec.tnmi7-0045-2017 PMC11687487 28429679

[7] Ben Salah I, Cayrou C, Raoult D, Drancourt M. 2009. Mycobacterium marseillense sp. nov., Mycobacterium timonense sp. nov. and Mycobacterium bouchedurhonense sp. nov., members of the Mycobacterium avium complex. Int J Syst Evol Microbiol 59:2803–2808. doi:10.1099/ijs.0.010637-0 19628609

[8] Daley CL, Iaccarino JM, Lange C, Cambau E, Wallace RJ Jr, Andrejak C, Böttger EC, Brozek J, Griffith DE, Guglielmetti L, Huitt GA, Knight SL, Leitman P, Marras TK, Olivier KN, Santin M, Stout JE, Tortoli E, van Ingen J, Wagner D, Winthrop KL. 2020. Treatment of nontuberculous mycobacterial pulmonary disease: an official ATS/ERS/ESCMID/IDSA clinical practice guideline. Eur Respir J 56:2000535. doi:10.1183/13993003.00535-2020 32636299 PMC8375621

[9] Palomino JC, Martin A. 2014. Drug resistance mechanisms in Mycobacterium tuberculosis. Antibiotics (Basel) 3:317–340. doi:10.3390/antibiotics3030317 27025748 PMC4790366

[10] Adachi Y, Tsuyuguchi K, Kobayashi T, Kurahara Y, Yoshida S, Kagawa T, Hayashi S, Suzuki K. 2020. Effective treatment for clarithromycin-resistant Mycobacterium avium complex lung disease. J Infect Chemother 26:676–680. doi:10.1016/j.jiac.2020.02.008 32171660

[11] Dubé MP, Sattler FR, Torriani FJ, See D, Havlir DV, Kemper CA, Dezfuli MG, Bozzette SA, Bartok AE, Leedom JM, Tilles JG, McCutchan JA. 1997. A randomized evaluation of ethambutol for prevention of relapse and drug resistance during treatment of Mycobacterium avium complex bacteremia with clarithromycin-based combination therapy. California Collaborative Treatment Group. J Infect Dis 176:1225–1232. doi:10.1086/514116 9359722

[12] Kemper CA, Havlir D, Haghighat D, Dubé M, Bartok AE, Sison JP, Yao Y, Yangco B, Leedom JM, Tilles JG. 1994. The individual microbiologic effect of three antimycobacterial agents, clofazimine, ethambutol, and rifampin, on Mycobacterium avium complex bacteremia in patients with AIDS. J Infect Dis 170:157–164. doi:10.1093/infdis/170.1.157 8014492

[13] van Ingen J, Egelund EF, Levin A, Totten SE, Boeree MJ, Mouton JW, Aarnoutse RE, Heifets LB, Peloquin CA, Daley CL. 2012. The pharmacokinetics and pharmacodynamics of pulmonary Mycobacterium avium complex disease treatment. Am J Respir Crit Care Med 186:559–565. doi:10.1164/rccm.201204-0682OC 22744719

[14] Maurer FP, Pohle P, Kernbach M, Sievert D, Hillemann D, Rupp J, Hombach M, Kranzer K. 2019. Differential drug susceptibility patterns of Mycobacterium chimaera and other members of the Mycobacterium avium-intracellulare complex. Clin Microbiol Infect 25:379. doi:10.1016/j.cmi.2018.06.010 29906595

[15] Zimenkov D. 2022. Variability of Mycobacterium avium complex isolates drug susceptibility testing by broth microdilution. Antibiotics (Basel) 11:1756. doi:10.3390/antibiotics11121756 36551413 PMC9774755

[16] Kwon BS, Kim MN, Sung H, Koh Y, Kim WS, Song JW, Oh YM, Lee SD, Lee SW, Lee JS, Lim CM, Choi CM, Huh JW, Hong SB, Park S, Shim TS, Chong YP, Jo KW. 2018. In vitro MIC values of rifampin and ethambutol and treatment outcome in Mycobacterium avium complex lung disease. Antimicrob Agents Chemother 62:e00491-18. doi:10.1128/AAC.00491-18 30012759 PMC6153803

[17] Telenti A, Philipp WJ, Sreevatsan S, Bernasconi C, Stockbauer KE, Wieles B, Musser JM, Jacobs WR Jr. 1997. The emb operon, a gene cluster of Mycobacterium tuberculosis involved in resistance to ethambutol. Nat Med 3:567–570. doi:10.1038/nm0597-567 9142129

[18] Sharma K, Gupta M, Pathak M, Gupta N, Koul A, Sarangi S, Baweja R, Singh Y. 2006. Transcriptional control of the mycobacterial embCAB operon by PknH through a regulatory protein, EmbR, in vivo. J Bacteriol 188:2936–2944. doi:10.1128/JB.188.8.2936-2944.2006 16585755 PMC1446986

[19] Safi H, Lingaraju S, Amin A, Kim S, Jones M, Holmes M, McNeil M, Peterson SN, Chatterjee D, Fleischmann R, Alland D. 2013. Evolution of high-level ethambutol-resistant tuberculosis through interacting mutations in decaprenylphosphoryl-β-D-arabinose biosynthetic and utilization pathway genes. Nat Genet 45:1190–1197. doi:10.1038/ng.2743 23995136 PMC6103293

[20] He L, Wang X, Cui P, Jin J, Chen J, Zhang W, Zhang Y. 2015. ubiA (Rv3806c) encoding DPPR synthase involved in cell wall synthesis is associated with ethambutol resistance in Mycobacterium tuberculosis. Tuberculosis (Edinb) 95:149–154. doi:10.1016/j.tube.2014.12.002 25547657

[21] Belanger AE, Besra GS, Ford ME, Mikusová K, Belisle JT, Brennan PJ, Inamine JM. 1996. The embAB genes of Mycobacterium avium encode an arabinosyl transferase involved in cell wall arabinan biosynthesis that is the target for the antimycobacterial drug ethambutol. Proc Natl Acad Sci USA 93:11919–11924. doi:10.1073/pnas.93.21.11919 8876238 PMC38159

[22] Moon SM, Kim SY, Kim DH, Huh HJ, Lee NY, Jhun BW. 2022. Relationship between resistance to ethambutol and rifampin and clinical outcomes in Mycobacterium avium complex pulmonary disease. Antimicrob Agents Chemother 66:e0202721. doi:10.1128/aac.02027-21 35266825 PMC9017369

[23] Litvinov V, Makarova M, Kudlay D, Nikolenko N, Mikhailova J. 2021. In vitro activity of bedaquiline against Mycobacterium avium complex. J Med Microbiol 70. doi:10.1099/jmm.0.001439 34668850

[24] Chen Y, Chen J, Zhang S, Shi W, Zhang W, Zhu M, Zhang Y. 2018. Novel mutations associated with clofazimine resistance in Mycobacterium abscessus. Antimicrob Agents Chemother 62. doi:10.1128/AAC.00544-18 PMC6021651 29712660

[25] Kong D, Kunimoto DY. 1995. Secretion of human interleukin 2 by recombinant Mycobacterium bovis BCG. Infect Immun 63:799–803. doi:10.1128/iai.63.3.799-803.1995 7868249 PMC173073

[26] Stover CK, de la Cruz VF, Fuerst TR, Burlein JE, Benson LA, Bennett LT, Bansal GP, Young JF, Lee MH, Hatfull GF. 1991. New use of BCG for recombinant vaccines. Nature 351:456–460. doi:10.1038/351456a0 1904554

[27] Huang H, Scherman MS, D’Haeze W, Vereecke D, Holsters M, Crick DC, McNeil MR. 2005. Identification and active expression of the Mycobacterium tuberculosis gene encoding 5-phospho-{α}-D-ribose-1-diphosphate: decaprenyl-phosphate 5-phosphoribosyltransferase, the first enzyme committed to decaprenylphosphoryl-D-arabinose synthesis. J Biol Chem 280:24539–24543. doi:10.1074/jbc.M504068200 15878857

[28] Zhang L, Zhao Y, Gao Y, Wu L, Gao R, Zhang Q, Wang Y, Wu C, Wu F, Gurcha SS, et al.. 2020. Structures of cell wall arabinosyltransferases with the anti-tuberculosis drug ethambutol. Science 368:1211–1219. doi:10.1126/science.aba9102 32327601

[29] Takayama K, Kilburn JO. 1989. Inhibition of synthesis of arabinogalactan by ethambutol in Mycobacterium smegmatis. Antimicrob Agents Chemother 33:1493–1499. doi:10.1128/AAC.33.9.1493 2817850 PMC172689

[30] Sreevatsan S, Stockbauer KE, Pan X, Kreiswirth BN, Moghazeh SL, Jacobs WR, Telenti A, Musser JM. 1997. Ethambutol resistance in Mycobacterium tuberculosis: critical role of embB mutations. Antimicrob Agents Chemother 41:1677–1681. doi:10.1128/AAC.41.8.1677 9257740 PMC163984

[31] Starks AM, Gumusboga A, Plikaytis BB, Shinnick TM, Posey JE. 2009. Mutations at embB codon 306 are an important molecular indicator of ethambutol resistance in Mycobacterium tuberculosis. Antimicrob Agents Chemother 53:1061–1066. doi:10.1128/AAC.01357-08 19104018 PMC2650568

[32] Nan X, Li M, Xiao T, Liu H, Lin S, Wang W, Qian C, Hang H, Li G, Zhao X, Wan K-L, Zhao L. 2024. Different contributions of embB and ubiA mutations to variable level of ethambutol resistance in Mycobacterium tuberculosis Isolates. Infect Drug Resist 17:3125–3132. doi:10.2147/idr.s466371 39050826 PMC11268779

[33] Lingaraju S, Rigouts L, Gupta A, Lee J, Umubyeyi AN, Davidow AL, German S, Cho E, Lee JI, Cho SN, Kim CT, Alland D, Safi H. 2016. Geographic differences in the contribution of ubiA mutations to high-level ethambutol resistance in Mycobacterium tuberculosis. Antimicrob Agents Chemother 60:4101–4105. doi:10.1128/AAC.03002-15 27139478 PMC4914663

[34] Xu Y, Jia H, Huang H, Sun Z, Zhang Z. 2015. Mutations found in embCAB, embR, and ubiA genes of ethambutol-sensitive and -resistant Mycobacterium tuberculosis clinical isolates from China. Biomed Res Int 2015:951706. doi:10.1155/2015/951706 26417605 PMC4568347

[35] Tulyaprawat O, Chaiprasert A, Chongtrakool P, Suwannakarn K, Ngamskulrungroj P. 2019. Association of ubiA mutations and high-level of ethambutol resistance among Mycobacterium tuberculosis Thai clinical isolates. Tuberculosis (Edinb) 114:42–46. doi:10.1016/j.tube.2018.11.006 30711156

[36] Giri A, Gupta S, Safi H, Narang A, Shrivastava K, Kumar Sharma N, Lingaraju S, Hanif M, Bhatnagar A, Menon B, Alland D, Varma-Basil M. 2018. Polymorphisms in Rv3806c (ubiA) and the upstream region of embA in relation to ethambutol resistance in clinical isolates of Mycobacterium tuberculosis from North India. Tuberculosis (Edinb) 108:41–46. doi:10.1016/j.tube.2017.10.003 29523326

[37] Lin S, Hua W, Wang S, Zhang Y, Chen X, Liu H, Shao L, Chen J, Zhang W. 2022. In vitro assessment of 17 antimicrobial agents against clinical Mycobacterium avium complex isolates. BMC Microbiol 22:175. doi:10.1186/s12866-022-02582-2 35804298 PMC9264595

[38] Zhao Y, Xu S, Wang L, Chin DP, Wang S, Jiang G, Xia H, Zhou Y, Li Q, Ou X, Pang Y, Song Y, Zhao B, Zhang H, He G, Guo J, Wang Y. 2012. National survey of drug-resistant tuberculosis in China. N Engl J Med 366:2161–2170. doi:10.1056/NEJMoa1108789 22670902

[39] Jin C, Wu Y, Chen J, Liu J, Zhang H, Qian Q, Pang T. 2024. Prevalence and patterns of drug-resistant Mycobacterium tuberculosis in newly diagnosed patients in China: a systematic review and meta-analysis. J Glob Antimicrob Resist 38:292–301. doi:10.1016/j.jgar.2024.05.018 38825149

[40] CLSI. 2023. Performance standards for susceptibility testing of mycobacteria, Nocardia spp., and other aerobic actinomycetes. 2nd ed. CLSI Supplement M24S. Clinical and Laboratory Standards Institude. 31339680

[41] Lian E, Belardinelli JM, De K, Pandurangan AP, Angala SK, Palčeková Z, Grzegorzewicz AE, Bryant JM, Blundell TL, Parkhill J, Floto RA, Wheat WH, Jackson M. 2025. Cell envelope polysaccharide modifications alter the surface properties and interactions of Mycobacterium abscessus with innate immune cells in a morphotype-dependent manner. mBio 16:e0032225. doi:10.1128/mbio.00322-25 40084888 PMC11980365

